# Micronutrient-fortified infant cereal improves Hb status and reduces iron-deficiency anaemia in Indian infants: an effectiveness study

**DOI:** 10.1017/S0007114519003386

**Published:** 2020-04-14

**Authors:** Shally Awasthi, Narayan U. Reddy, Monjori Mitra, Shweta Singh, Sanjeev Ganguly, Ivana Jankovic, Dominik Grathwohl, Colin I. Cercamondi, Apurba Ghosh

**Affiliations:** 1 Department of Pediatrics, King George’s Medical University, Lucknow, Uttar Pradesh 226003, India; 2 Princess Esra Hospital, Deccan College of Medical Sciences & Allied Hospitals, Hyderabad, Telangana 500002, India; 3 Department of Pediatrics, Institute of Child Health, Kolkata 700017, India; 4 Department of Psychiatry, King George’s Medical University, Lucknow, Uttar Pradesh 226003, India; 5 Global Medical Affairs, Société des Produits Nestlé S.A., 1800 Vevey, Switzerland; 6 Nestlé Product Technology Center, Nutrition, Société des Produits Nestlé S.A., 1800 Vevey, Switzerland; 7 Nestlé Research, Société des Produits Nestlé S.A., 1000 Lausanne, Switzerland

**Keywords:** Infants, Fortified infant cereal, Anaemia, Iron deficiency, Hb, Neurodevelopment, Bayley Scales of Infant and Toddler Development Third Edition scores

## Abstract

Anaemia affects approximately 69 % of Indian children aged 6–12 months, with Fe deficiency (ID) being a common cause. The effectiveness of micronutrient-fortified infant cereal in improving Fe status and neurodevelopment was evaluated in non-anaemic and mildly anaemic Indian infants. An intervention group (IC) enrolled at age 6 months consumed 50 g/d of rice-based cereal providing 3·75 mg Fe/d as ferrous fumarate for 6 months (*n* 80) and was compared with a matched static cross-sectional control group (CG) without intervention enrolled at age 12 months (*n* 80). Mean Hb was higher in IC (118·1 (sd 10·2) g/l) *v.* CG (109·5 (sd 16·4) g/l) at age 12 months (adjusted mean difference: 9·7 g/l; 95 % CI 5·1, 14·3; *P* < 0·001), while geometric mean serum ferritin tended to be higher (27·0 (–1 sd 13·4, +1 sd 54·4) *v.* 20·3 (–1 sd 7·5, +1 sd 55·0) ng/ml); *P* = 0·085) and soluble transferrin receptor was lower (1·70 (–1 sd 1·19, +1 sd 2·43) *v.* 2·07 (–1 sd 1·29, +1 sd 3·33) mg/l; *P* = 0·014). Anaemia (23 *v.* 45 %; *P* = 0·007) and ID (17 *v.* 40 %; *P* = 0·003) were lower in IC *v.* CG. Bayley Scales of Infant and Toddler Development Third Edition scores for language (*P* = 0·003), motor development (*P* = 0·018), social-emotional (*P* = 0·004) and adaptive behaviour (*P* < 0·001), but not cognitive development (*P* = 0·980), were higher in IC *v.* CG. No significant difference in anthropometric *Z*-scores was observed between the groups. Consuming a micronutrient-fortified infant cereal daily for 6 months during complementary feeding promoted better Fe status while reducing the risk for anaemia and ID and was associated with superior neurodevelopmental scores.

Globally, the WHO estimates that 273 million young children are anaemic^(1)^, of which approximately 60 % is attributable to Fe-deficiency anaemia (IDA)^(2)^. Infants and toddlers between 6 and 24 months of age have the highest rates of IDA^(3)^. At approximately 6 months of age, the Fe stores accumulated *in utero* and the supply of highly bioavailable Fe from breast milk are no longer adequate to cover Fe requirements to support the rapid growth and development that continue in late infancy^(4)^. Consequently, if complementary foods (CF) do not provide adequate amounts of bioavailable Fe, the young child is at high risk to develop a continuum, starting with Fe deficiency (ID) and then IDA. In many lower-middle-income countries, such as India, CF consist of home-made non-fortified cereals or starchy roots and tubers, many of which have a low concentration and/or bioavailability of Fe and, hence, will not provide sufficient Fe to the growing child^(5)^. In India, the prevalence of anaemia in infants 6–12 months of age is estimated to be 69 %^(6)^ and is a major economic burden to the society^(7)^.

Infants with IDA are at risk for compromised cognitive, motor, social-emotional and neurophysiological development in the short and long term^(3)^. In several Fe intervention studies, early cognitive and/or motor development scores in Fe-deficient anaemic infants assessed at baseline were inferior compared with infants with better Fe status^(8–12)^. Fe supplementation for 3–6 months in Fe-deficient anaemic infants did improve developmental test scores in some of the aforementioned studies^(8,9)^ but not in the others^(10–12)^. Impaired cognitive and motor development may be irreversible or only partially reversible by the provision of Fe depending on the duration, severity and timing of IDA^(3,13)^. Longitudinal studies on long-term effects of IDA observed that children who had IDA in infancy did worse on tests of mental, motor and social-emotional functioning in later childhood and adolescence, despite Fe therapy and Fe status improvement^(3)^. A limited number of studies suggest a beneficial effect of Fe-fortified foods on short-term infant neurodevelopment^(14,15)^. Systematic reviews do not provide adequate evidence that Fe supplementation or fortification improves cognitive and motor development in the overall infant and young children population^(16–19)^; however, subgroup meta-analysis suggests a beneficial effect in Fe-deficient children^(16)^ and provides limited evidence for benefits on motor development in non-anaemic infants^(19)^.

A safe and cost-effective approach to alleviate nutritional ID in infants is Fe fortification of CF which can be done either commercially at the time of food production or through the addition of nutrients in the home before consumption (referred to as in-home fortification). Commercially fortified CF typically comprise milk or cereal products (e.g. porridge or gruel)^(20)^. Two systematic reviews concluded that micronutrient-fortified CF are an effective approach for providing additional dietary Fe and reducing anaemia rates^(21,22)^. However, most of the evidence comes from Fe-fortified milk products, and only a few studies are available investigating the efficacy of fortified infant cereals in improving Fe status. In a randomised controlled trial (RCT), ID prevalence was reduced in Ghanaian infants receiving a micronutrient-fortified cereal-legume blend fed *ad libitum* and providing 12–18 mg additional Fe as electrolytic Fe compared with a control group but did not improve Hb concentration or reduce anaemia^(23)^. Similarly, micronutrient-fortified rusk providing 5 mg additional Fe as ferric ammonium citrate had only a small significant benefit on Hb concentration in Chinese infants^(24)^. RCT using ferrous fumarate (FeF) as an Fe compound showed stronger effect on Fe status with higher Hb and serum ferritin (SF) concentration and reduced anaemia and ID in infants receiving additional 5·5 or 12·5 mg Fe/d from a micronutrient-fortified maize porridge compared with their control peers^(14,25)^. However, in the light of evidence that higher doses of Fe lead to a range of adverse events in low-income settings^(26)^, there is a need to test lower doses of FeF. Limited data are currently available on the impact of lower doses of FeF of less than 5 mg Fe/d. In Chinese infants, a low dose of approximately 1 mg additional Fe/d as FeF from a multi-fortified infant cereal fed for 12 months showed marginally improved SF concentrations and had no effect on Hb^(27)^. Further evidence on low to moderate doses of FeF is needed. Therefore, the aim of the present study was to generate data on the effectiveness of a micronutrient-fortified rice-based infant cereal providing a low to moderate dose of additional 3·75 mg Fe/d as FeF in promoting Fe status as well as investigating the effect on neurodevelopment in Indian infants. The study was designed with a longitudinal intervention group (IC) where infants were enrolled at the age of 6 months and followed up until 12 months of age, and a matched static cross-sectional control group (CG) of infants who did not receive the intervention and who were only enrolled and assessed at the age of 12 months. We hypothesised that infants in the IC would have better Fe status and more favourable neurodevelopmental outcomes than the infants in the CG group at 12 months of age.

## Methods

### Study design and population

**Fig. 1.**
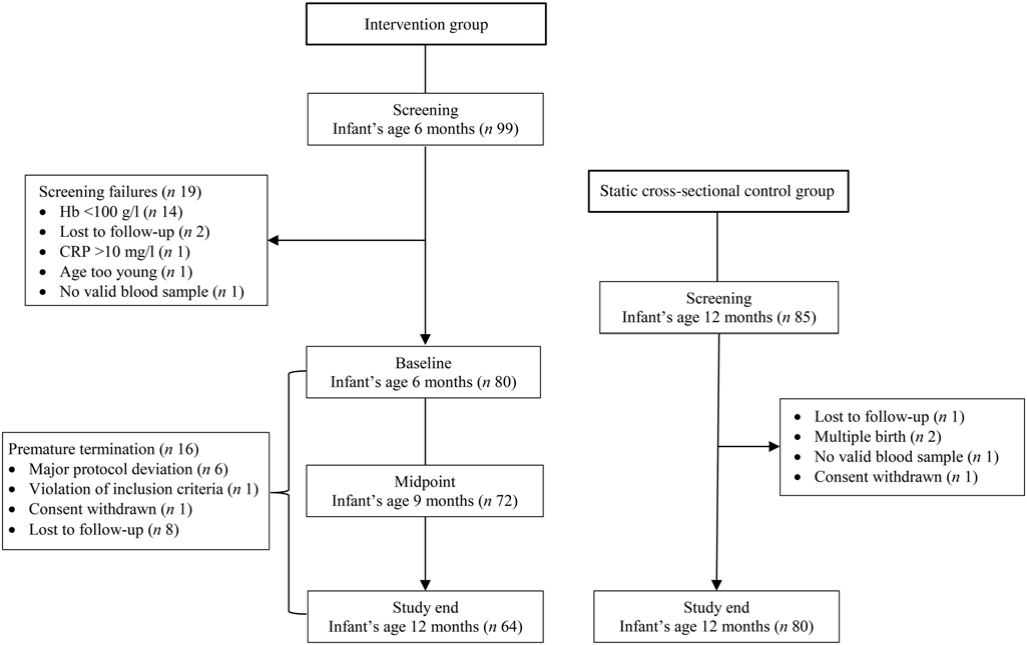
Study design and participant flow chart. CRP, C-reactive protein.

This multi-center, open-label intervention trial with a matching static cross-sectional CG was conducted between April 2017 and May 2018 in three sites in India: King George’s Medical University (Lucknow), Princess Esra Hospital (Hyderabad) and Institute of Child Health (Kolkata). The study population included a total of 160 healthy infants aged 6 and 12 months, who were recruited simultaneously for IC (*n* 80) or CG (*n* 80) (Fig. 1) when they had their routine vaccination visits or well-being check-ups at the sites. Apparently healthy, single-birth infants who were 6 months of age with normal weight-for-age within ±2 sd of WHO growth standards and who had CF introduced were eligible for the IC. Infants who had (1) chronic, acute ongoing or recent (last 2 weeks) illness necessitating medical follow-up, (2) parasitic infections, (3) documented intolerance to gluten or lactose, (4) documented allergy to bovine milk proteins, (5) severe or moderate anaemia (Hb < 100 g/l), (6) inflammation as indicated by elevated C-reactive protein (CRP) >10 mg/l and (7) participated in another clinical study ≤4 weeks prior to enrolment were excluded from the IC. Apparently healthy, 12-month-old single-birth infants, who did not have chronic, acute ongoing or recent (last 2 weeks) illness necessitating medical follow-up and did not participate in another clinical study ≤4 weeks prior to enrolment, were enrolled in the CG. These infants served as a static cross-sectional control group. With this design, we avoided any potential concerns of enrolling anaemic and/or Fe-deficient infants or infants that are in general at high risk for ID into a control group. A simple matching approach based on the close monitoring of five selected key demographic characteristics (sex, birth weight, delivery mode, gestational age and milk feeding pattern) in the already enrolled infants in IC was attempted to match infants in CG with infants in IC.

The study was conducted according to the guidelines laid down in the Declaration of Helsinki, and the protocol was approved by the Institutional Ethics Committees of the King George Medical University in Lucknow, the Institute of Child Health in Kolkata and the Deccan College of Medical Sciences & Allied Hospitals in Hyderabad. Written informed consent was obtained from the parents or legally authorised representatives. The study was registered prospectively with Clinical Trials Registry-India (CTRI/2017/02/007767).

### Intervention and study product

Within 2 weeks of initial screening visit, infants in the IC had a baseline visit after which they received 50 g/d of a fortified cereal (Société des Produits Nestlé S.A.) throughout the 6 months of intervention in addition to the habitual complementary feeding regimen. The nutritional composition of the micronutrient-fortified rice-based infant cereal is presented in Table 1. The infant cereal provided 75 % (3·75 mg) of the Indian daily recommended intake (DRI) for Fe in infants (5 mg)^(28)^ so that other CF or breast milk could still contribute to the DRI. Depending on the infant’s age and feeding behaviour, caregivers were instructed to feed 50 g of infant cereal either as one serving or as two servings of 25 g. Caregivers were trained to reconstitute 25/50 g infant cereal powder with 75/150 ml of lukewarm water in order to obtain a purée consistency allowing to feed the infant cereal with a spoon. Post-baseline assessments in the IC were conducted at midpoint (9 months of age) and at study end (12 months of age). Infants in the CG were enrolled at 12th month of life at the screening visit, and they had the study end visit within 2 weeks of the screening visit. The CG was considered a static cross-sectional non-interventional control.

**Table 1.** Energy and nutrient composition of the rice-based micronutrient-fortified infant cereal consumed for 6 months by the infants in the intervention group

Nutritional component	Amount/daily serving
Energy (kJ)	868
Protein (g)	7·5
Total fat (g)	4·5
Linoleic acid (g)	0·75
*α*-Linolenic acid (mg)	92·5
Carbohydrate (g)	34·25
Sugar (g)	4·5
Fibre (g)	0·25
Na (mg)	57·5
Ca (mg)	219
K (mg)	175
P (mg)	175
Mg (mg)	22·5
Fe (mg)	3·75
Cu (mg)	0·15
Zn (mg)	1·25
Vitamin A (µg)	175
Vitamin D (µg)	2·5
Vitamin E (mg)	1·25
Vitamin C (mg)	18·75
Thiamin (mg)	0·25
Riboflavin (mg)	0·185
Niacin (mg)	2·75
Pantothenic acid (mg)	0·75
Vitamin B_6_ (mg)	0·2
Folic acid (µg)	12·5
Biotin (µg)	2·6
Vitamin B_12_ (µg)	0·1

### Socio-demographic, anthropometric, morbidity, safety and dietary outcomes

To assess subject eligibility during screening, socio-demographic characteristics and child medical history data were collected via questionnaires and anthropometric measurements were taken in both groups. In the IC, anthropometric measurements were also done at midpoint and study end. Infant weight was recorded using a calibrated digital baby scale (Seca 354 M) and length using a calibrated paediatric measuring board (Seca 417). Furthermore, head circumference was measured using a flexible measuring tape (Seca disposable measuring tape 211). Weight-for-age, length-for-age, weight-for-length and head circumference-for-age *Z*-scores were calculated using WHO reference data^(29)^. Concomitant medications, including the prospective use of Fe supplements based on a low Hb concentration (<100 g/l) during a study visit, were monitored throughout the entire study. Morbidity and safety were assessed from (1) a sickness questionnaire at baseline, midpoint and study end visit for IC and at study end visit for CG and (2) recording of adverse events (AE) collected through infants’ diary cards during each visit for both groups. To make outcomes on AE and concomitant medications comparable between the two study groups (i.e. to control for the different amounts of time, each group was in the study), an incidence rate for AE and for concomitant medications was calculated as follows: Total number of AE or medications reported among all enrolled subjects divided by the sum of length of duration (in days) for all subjects that were on the respective treatment arms multiplied by 100. Nutrient intake was evaluated using a 24-h dietary recall administered by a trained paediatric dietitian to the infant’s parent or caregiver at study end visits for the IC and CG. Breast milk intake during the 24-h dietary recall was assessed as frequency of breast milk feeds. The daily intake of energy, macronutrients (fat, carbohydrates and protein) and selected micronutrients (Fe, Zn, Cu, Mg, vitamins A, B_12_, C, D and folate) coming from CF at 12 months of age (not including nutrients from breast milk) was calculated using Indian Food Composition Tables^(30)^. Quality of life losses due to impaired cognitive development as Disability Adjusted Life Years and the production losses based on the Hb difference in our study were calculated using data from the most recent National Family Health Survey in India^(6)^ and the methodology previously described by Plessow *et al*.^(7)^ not including severely anaemic infants.

### Haematological outcomes

Blood samples were taken at screening (6 months of age), midpoint (9 months of age) and study end (12 months of age) in the IC and at the single time point during the screening in the CG (12 months of age). Non-fasting peripheral venepuncture blood samples were drawn into anticoagulant-coated tubes (Becton Dickinson) for Hb assessment and into plain tubes (Becton Dickinson) for SF, serum soluble transferrin receptor (sTfR) and CRP analysis. After collection, whole blood for SF, sTfR and CRP analysis was allowed to clot at room temperature followed by centrifugation to separate the serum. The respective serum containers were stored at refrigerated condition until assessment. Hb (primary end point) was measured at screening, midpoint and study end in the IC and at screening only in the CG using automated haematology analysers (Beckman coulter DXH – 800/Sysmex XN 1000). SF, sTfR and CRP (secondary end points) were determined at screening and at study end visit in the IC and at study end visit only in the CG. SF was measured by chemiluminescent immunoassay system (CMIA-ABBOTT ARCHITECT i2000). CRP and sTfR were evaluated by nephelometry (Nephelometry-Siemens BN II). Anaemia was defined as Hb < 110 g/l. Moderate and severe anaemia were defined as Hb > 70 but <100 g/l and Hb <70 g/l, respectively. In both groups, infants with Hb < 100 g/l at any of the study time points received Fe supplements as per local medical standards. In the IC, infants with Hb < 100 g/l at screening or midpoint (major protocol deviation) were excluded or withdrawn from the study, respectively. ID was defined as SF < 12 μg/l and CRP < 5 mg/l or SF < 30 μg/l and CRP ≥ 5 mg/l; IDA was defined as Hb <110 g/l and SF < 12 μg/l and CRP < 5 mg/l or Hb <110 g/l and SF < 30 μg/l and CRP ≥ 5 mg/l^(4)^.

### Infant neurodevelopmental outcomes

Neurodevelopment was assessed using the Bayley Scales of Infant and Toddler Development Third Edition (Bayley-III)^(31)^ at baseline and study end in the IC (6 and 12 months of age) and at study end (12 months of age) in the CG. The Bayley-III is a standardised test for 1- to 42-month-old children assessing age-appropriate skills in five developmental scales: cognitive, language, motor, social-emotional and adaptive behaviour. Cognitive, language and motor skills were tested by certified medical staff. To complete the questionnaires on the social-emotional and adaptive behaviour development, caregivers were assisted by the certified medical staff. An age-corrected composite score was derived for each of the five scales and scaled to a metric, with a mean of 100, a standard deviation of 15 and a range of 40–160^(31)^.

### Statistical analysis

A sample size of approximately fifty infants (twenty-five per study group) was required to detect an expected difference of 8·0 g/l in Hb concentration between the two study groups at 12 months of age, with a statistical power of 90 % and a two-sided significance level of 0·05. The assumed standard deviation for Hb concentration was 8·5 g/l^(4)^. Nonetheless, eighty infants per arm were recruited to account for an anticipated drop-out rate of 30 % and to have adequate power for secondary end points.

Data analysis was conducted in the analysis set (IC *n* 64; CG *n* 80), which was defined as infants having a valid blood sample at 12 months of age who did not require treatment with high-dose Fe supplements at 9 months of age due to the development of moderate or severe anaemia, using the software Statistical Analysis System (SAS, version 9.3 or higher). Descriptive and demographic characteristics used for the matching were compared between IC and CG using Pearson’s *χ* ^2^ or Fisher’s exact test for categorical data and independent *t* test for continuous data. Fe status assessed by Hb, SF and sTfR concentrations, inflammation measured as CRP and Bayley-III scores between IC and CG were compared using ANCOVA models correcting for sex, exclusively breast-feeding until 6 months of age (yes/no), Kuppuswamy socio-economic status^(32)^, birth weight, gestational age and study site. Anthropometric parameters were compared between the two study groups using ANCOVA models correcting for sex, birth weight, gestational age and study site. Pearson’s *χ* ^2^ test was used for the comparison of anaemia, ID and IDA prevalence between IC and CG at 12 months of age. Changes in the Hb concentration (primary end point) within the IC over time from baseline (6 months of age) to study end (12 months of age), baseline to midpoint (9 month of age) and midpoint to study end were analysed using paired *t* test. Frequency of breast-feeding, energy and nutrient intakes between the two study groups was compared by independent *t* test. For not normally distributed data as checked by Q–Q plots (SF, sTfR, CRP, certain nutrient intakes), tests were done on log-transformed data. All statistical tests were conducted using a two-sided test at an *α* = 0·05 level of significance. Standardised effect size between the two groups in Bayley-III scores was estimated by Cohen’s *d* estimate, wherein *d* values of 0·2, 0·5 and 0·8 represent small, medium and large effect sizes, respectively^(33)^.

## Results

### Participant characteristics

A total of ninety-nine and eighty-five infants were screened for enrolment in the IC and CG, respectively, and eighty infants were recruited in each group (Fig. 1). During the intervention, sixteen infants dropped out in the IC and sixty-four infants completed the study. All eighty infants included in the CG completed the study.

Maternal and infant characteristics at screening are summarised in Table 2 and were comparable between the two groups. There were no statistical differences between the groups in the ratio of boys:girls, mean birth weight, gestational age and mode of delivery. The delivery mode in both groups was predominately by caesarean section. The majority of the infants in IC (78 %) were exclusively breast-fed before enrolment at 6 months of age. These were slightly less infants than in CG (*P* = 0·003) where 94 % were exclusively breast-fed before other foods were introduced at a mean age of 6·6 (sd 1·3) years. In the IC, foods were introduced at a mean age of 5·6 (sd 1·3) years. The socio-economic status based on Kuppuswamy’s scale^(32)^ showed that the majority of the infants in both groups were from the upper-lower or lower socio-economic strata. The participating mothers in both groups were predominantly high school graduates (31 % for IC *v.* 30 % for CG), non-employed (94 % for IC *v.* 99 % for CG) and living in urban or suburban locations (99 % for IC *v.* 94 % for CG).

**Table 2.** Demographic infant and maternal characteristics in the intervention (IC) and static cross-sectional control group (CG) at enrolment (Mean values and standard deviations; percentages)

Characteristics	IC (*n* 80)			CG (*n* 80)			*P* *
	Mean		sd	Mean		sd	
Infant characteristics							
Age at enrolment (months)†	5·7		0·5	11·8		0·4	
Gestational age at birth (weeks)	36·6		0·9	36·6		0·8	0·853‡
Sex (% boys)		46·3			52·5		0·429§
Birth weight (kg)	2·9		0·5	2·8		0·5	0·425‡
Delivery mode (% Caesarean section)		65·0			61·3		0·623§
Exclusively breast-fed up to 6 months of age (%)		77·5			93·8		0·003‖
Maternal characteristics							
Main caregiver (% mother)		100			100		
Maternal age (years)	27·3		4·3	26·4		3·8	
Parity (number)	1·8		1·0	1·8		0·8	
Level of education (%)							
Unschooled/primary or middle school certificate		28·7			23·7		
High school certificate/intermediate or post high school diploma		42·5			43·8		
Graduate/postgraduate/doctorate/PhD		28·8			32·5		
Employment status (% non-employment)		93·8			98·8		
Living setting (%)							
Urban		85·0			75·0		
Sub-urban		13·8			18·8		
Rural		1·2			6·2		
Socio-economic status (%)¶							
Upper/upper middle		16·2			15·0		
Lower middle		22·5			27·5		
Upper lower/lower		61·3			57·5		

* *P* value between group comparison; only assessed for the characteristics that were used as matching criteria between infants in the IC and CG.

† Infants in the IC were enrolled at approximately 6 months of age; infants in the CG at approximately 12 months of age.

‡ Independent *t* test for between-group comparison.

§ Pearson’s *χ* ^2^ test for between-group comparison.

‖ Fisher’s exact test for between-group comparison.

¶ Socio-economic status was based on Kuppuswamy’s scale^(32)^.

### Iron status

**Fig. 2.**
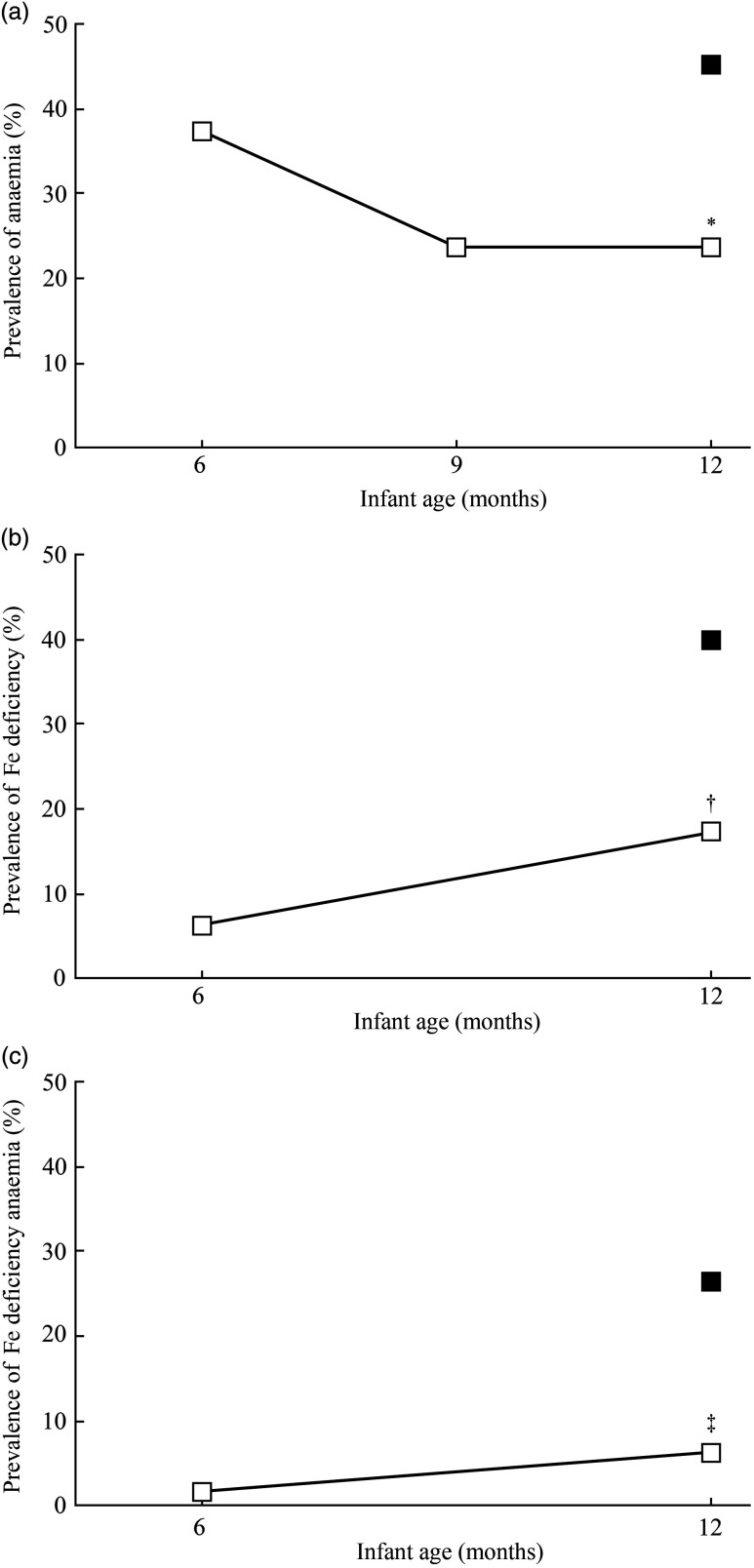
Prevalence of anaemia (a), iron deficiency (b) and iron-deficiency anaemia (c) at 6 and 12 months of age for infants in the intervention group (IC; *n* 64) and at 12 months of age for infants in the static cross-sectional control group (CG; *n* 80). 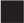, CG; 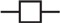, IC. Significantly different from CG using Pearson’s *χ* ^2^ test: * *P* = 0·007; † *P* = 0·003; ‡ *P* = 0·002. Anaemia defined as Hb <110 g/l; iron deficiency defined as serum ferritin <12 μg/l and C-reactive protein (CRP) < 5 mg/l or serum ferritin <30 μg/l and CRP ≥ 5 mg/l; iron-deficiency anaemia defined as Hb <110 g/l and serum ferritin <12 μg/l and CRP < 5 mg/l or Hb <110 g/l and serum ferritin <30 μg/l and CRP ≥ 5 mg/l.

Table 3 shows the Fe status parameters and CRP concentration at 6 and 12 months of age for the IC and at 12 months of age only for the CG. At 12 months of age, four infants in each group had elevated CRP concentration (>10 mg/l). At 12 months of age, compared with the CG, Hb concentration was higher in the IC (adjusted mean difference: 9·7 g/l; 95 % CI 5·1, 14·3; *P* < 0·001), whereas sTfR concentration was lower in the IC (–18·1 %; 95 % CI –32·4, –3·7; *P* = 0·014). Compared with the CG, the SF concentration in the IC at 12 months of age tended to be higher (26·6 %; 95 % CI –3·7, 56·8; *P* = 0·085). Hb concentration significantly increased from 6 to 12 months of life in the IC (*P* < 0·001), with a Hb concentration at midpoint of 115·9 (sd 9·9) g/l. In the IC, a trend for a significant increase in Hb concentration between 6 and 9 months of age (*P* = 0·081) and a non-significant increase from 9 to 12 months of life was observed (*P* = 0·116). In the sixteen infants, who dropped out in IC, mean Hb was 111·0 (sd 7·4) g/l, geometric mean SF was 29·2 (–1 sd 9·6, +1 sd 89·1) and geometric mean sTfR was 1·95 (–1 sd 1·54, +1 sd 2·46) at baseline. Fig. 2 shows that at 12 months of age, the prevalence of anaemia in IC was 49 % lower than that in CG (23 % for IC *v.* 45 % for CG; *P* = 0·007) (Fig. 2(a)), ID was 58 % lower (17 % for IC *v.* 40 % for CG; *P* = 0·003) (Fig. 2(b)) and IDA was 77 % lower (6 % for IC *v.* 26 % for CG; *P* = 0·002) (Fig. 2(c)). At 12 months of age, 22 % of the infants in the IC had mild anaemia (Hb >100 but <110 g/l) and 1 % had moderate anaemia (Hb >70 but <100 g/l). In the CG, the prevalence of mild and moderate anaemia at 12 months of age was 19 and 26 %, respectively.

**Table 3.** Iron status indices and C-reactive protein concentration of infants in the intervention group at 6 and 12 months of age (IC) and in the static cross-sectional control group (CG) at 12 months of age (Mean values and standard deviations; geometric mean and –1 standard deviation and +1 standard deviation)

Parameter	IC (*n* 64)						CG (*n* 80)			*P* *
	6 months			12 months			12 months			
	Geometric mean		–1 sd, +1 sd	Geometric mean		–1 sd, +1 sd	Geometric mean		–1 sd, +1 sd	
Hb (g/l)										
Mean		113·4			118·1			109·5		<0·001
sd		8·7			10·2			16·4		
Serum ferritin (ng/ml)	40·0		16·4, 78·7	27·0		13·4, 54·4	20·3		7·5, 55·0	0·085
Serum soluble transferrin receptor (mg/l)	1·68		1·22, 2·31	1·70		1·19, 2·43	2·07		1·29, 3·33	0·014
Serum C-reactive protein (mg/l)	0·46		0·16, 1·34	0·84		0·20, 3·60	0·77		0·17, 3·39	0·741

* *P* value for between-group comparison (ANCOVA model correcting for sex, exclusively breast-feeding until 6 months of age (yes/no), Kuppuswamy socio-economic status^(32)^, birth weight, gestational age and study site).

### Neurodevelopment

**Fig. 3.**
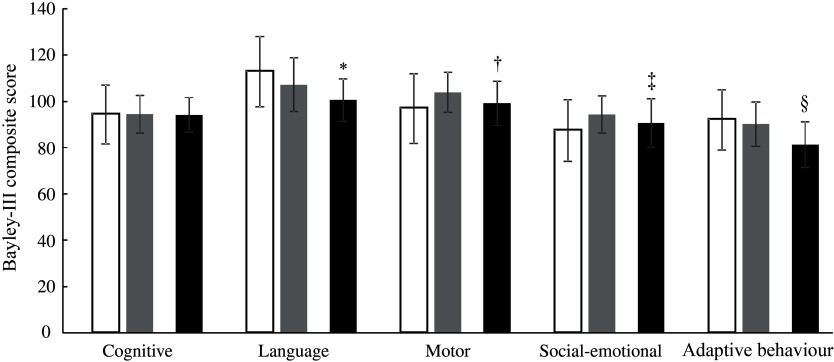
Neurodevelopment (Bayley Scales of Infant and Toddler Development Third Edition (Bayley-III) scores) of the infants in the intervention group at 6 and 12 months of age (IC; *n* 64) and in the static cross-sectional control group at 12 months of age (CG; *n* 80). 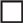, IC 6 months; 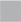, IC 12 months; 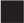, CG 12 months. *†‡§ *P* value (ANCOVA model correcting for sex, exclusively breast-feeding until 6 months of age (yes/no), Kuppuswamy socio-economic status^(32)^, birth weight, gestational age and study site). * Significantly different from IC 12 months (*P =* 0·001). † Significantly different from IC 12 months (*P* = 0·022). ‡ Significantly different from IC 12 months (*P* = 0·005). § Significantly different from IC 12 months (*P <* 0·001).

At 12 months of age, the Bayley-III score for language development was higher (adjusted mean difference: 5·3; 95 % CI 2·0, 8·5; *P* = 0·001) in the IC (107·2 (sd 11·6)) than in the CG (100·5 (sd 9·2)), and the motor development score was better (3·4; 95 % CI 0·5, 6·3; *P* = 0·022) in the IC (103·9 (sd 8·7)) compared with the CG (99·1 (sd 9·5)). Compared with the CG (90·6 (sd 10·5)), the social-emotional behaviour score in the IC (94·3 (sd 8·0)) was higher (4·2; 95 % CI 1·3, 7·1; *P* = 0·005), and the adaptive behaviour score was better (6·0; 95 % CI 3·7, 8·3; *P* < 0·001) in the IC (90·2 (sd 9·6)) than in the CG (81·3 (sd 9·9)) at 12 months of age (Fig. 3). The mean Bayley-III scores for cognition did not differ between the IC (94·4 (sd 8·2)) and the CG (94·1 (sd 7·5)) at 12 months of age (*P* = 0·982). Based on Cohen’s *d* estimate, the magnitude of difference (effect size) between the IC and CG was strong for adaptive behaviour (*d* = 0·91) and language development (*d* = 0·65) and medium for motor development (*d* = 0·53) and social-emotional behaviour (*d* = 0·39).

### Anthropometry

At 12 months of age, growth outcomes in the two groups were comparable as indicated by similar anthropometric *Z*-scores. *Z*-scores at 12 months of age for IC and CG, respectively, were (1) weight-for-age: –0·5 (sd 1·0) *v.* –0·3 (sd 0·8), *P* = 0·360; (2) length-for-age: –1·6 (sd 1·0) *v.* –1·3 (sd 1·4), *P* = 0·117; (3) weight-for-length: 0·4 (sd 1·1) *v.* 0·4 (sd 1·1), *P* = 0·931 and (4) head circumference-for-age: –0·9 (sd 1·2) *v.* –0·9 (sd 1·2), *P* = 0·424. In the IC at 6 months of age, *Z*-scores were (1) weight-for-age: –0·6 (sd 0·8); (2) length-for-age: –1·2 (sd 0·8); (3) weight-for-length: 0·2 (sd 0·9) and (4) head circumference-for-age: –0·3 (sd 1·5). In the IC at 9 months of age, *Z*-scores were (1) weight-for-age: –0·6 (sd 0·8); (2) length-for-age: –1·6 (sd 1·0); (3) weight-for-length: 0·4 (sd 0·9) and (4) head circumference-for-age: –0·8 (sd 1·4).

### Dietary outcomes

CF other than breast milk were provided to all sixty-four infants of the IC at 12 months of age and for all eighty infants of the CG. Mean daily energy intakes from CF were not different between the IC and the CG (2030 (sd 675) *v.* 1986 (sd 768) kJ/d, respectively; *P* = 0·714) at 12 months of age. The mean frequency of breast milk feeds was lower in the IC *v.* the CG group at 12 months of age (6·6 (sd 3·3) times/d *v.* 9·1 (sd 5·7) times/d; *P* = 0·002). As reported in Table 4, the intakes of total fat, linoleic acid and *α*-linoleic acid from CF were higher in the IC compared with the CG at 12 months of age (*P* < 0·001), while there was no difference for carbohydrate and protein intake between the groups (*P* > 0·05). Geometric mean intake of Fe from CF was approximately three times higher in the IC compared with the CG (*P* < 0·001). Similarly, intakes of Zn, Cu, Mg, vitamins A and C from CF were also higher in the IC than in the CG at 12 months of age (*P* < 0·001; except for Cu with *P* < 0·005). The vitamin B_12_ intake from CF in the IC tended to be higher than that in the CG (*P* = 0·053). Folate and vitamin D intake from CF was not different between the groups at 12 months of age (*P* > 0·05).

**Table 4.** Nutrient intake from complementary foods in the infants of the intervention group (IC) and in the static cross-sectional control group (CG) at 12 months of age using 24-h food recall (Mean values and standard deviations; geometric mean and –1 standard deviation and +1 standard deviation)

Nutrient	IC (*n* 64)			CG (*n* 80)			*P* *
	Geometric mean		–1 sd, +1 sd	Geometric mean		–1 sd, +1 sd	
Total fat (g/d)	8·0		4·5, 14·0	3·1		0·9, 10·3	<0·001
Linoleic acid (g/d)	3·9		1·4, 11·0	1·2		0·1, 12·7	<0·001
*α*-Linolenic acid (mg/d)	86·8		16·5, 455·6	7·5		1·1, 53·0	<0·001
Carbohydrate (g/d)							
Mean		66·0			69·5		0·420
sd		23·9			27·7		
Protein (g/d)							
Mean		18·1			17·4		0·590
sd		7·1			10·0		
Fe (mg/d)	4·6		3·4, 6·3	1·5		0·3, 6·8	<0·001
Zn (mg/d)	2·0		1·2, 3·4	1·2		0·4, 3·1	<0·001
Cu (mg/d)	0·3		0·2, 0·5	0·2		0·1, 0·6	0·002
Mg (mg/d)	42·7		23·6, 77·2	8·5		2·1, 35·4	<0·001
Vitamin A (µg/d)	209·6		153·3, 286·6	30·2		2·3, 389·9	<0·001
Vitamin B_12_ (µg/d)	0·18		0·01, 2·21	0·08		0·01, 0·72	0·053
Folate (µg/d)	18·7		1·5, 236·9	34·4		3·5, 334·8	0·137
Vitamin C (mg/d)	24·0		14·3, 40·2	2·4		0·6, 9·1	<0·001
Vitamin D (µg/d)	10·5		3·4, 32·1	6·4		0·9, 44·3	0·060

* *P* value from independent *t* test.

### Adverse events, morbidity, concomitant medication, and quality of life and production losses

After controlling for the shorter observational period in the CG (2 weeks only), the incidence rate of AE was 0·19 and 0·26 % in the IC and CG, respectively. Overall, twenty-eight AE were reported in twenty-two infants in the IC and four AE occurred in three infants in the CG. The AE reported in the CG included pyrexia (*n* 3) and nasopharyngitis (*n* 1). In the IC, nasopharyngitis (*n* 10), lower respiratory tract infection (*n* 1), pyrexia (*n* 8), diarrhoea (*n* 5), cough (*n* 3) and severe anaemia (*n* 1) were observed. There were no incidences of AE related to the studied infant cereal. The incidence rate of concomitant medication, controlling for the shorter observational period in the CG (2 weeks only), was 0·16 and 1·49 % in the IC and CG, respectively. Overall, twenty-four administrations of concomitant medications were reported in the IC and twenty-three administrations in the CG. Fe-containing supplements were more commonly required in the CG than in the IC. Twenty-one infants in CG had low Hb concentration <100 g/l at 12 months of age and needed Fe supplements, while only seven infants in IC needed Fe supplements due to low Hb at 9 months (*n* 6) and 12 months of age (*n* 1). In the IC, seven infants took analgesics, whereas only two infants took the same in the CG.

In our model on quality of life losses and production losses not including the severely anaemic infants, we found that a fortified infant cereal could save future production losses of 1353 million USD in India and approximately 313,000 Disability Adjusted Life Years, which correspond to thirteen complete lifespans saved in India every single day.

## Discussion

In our study using a static cross-sectional group comparison design, infants aged 6 months who received a multi-fortified rice-based cereal for 6 months had better Fe status at 12 months of age and lower rates of ID and IDA than infants who did not receive the study cereal. This indicates that the additional Fe from the study cereal helped the infants in the IC to significantly reduce the risk of developing ID and IDA by 58 and 77 %, respectively. In the IC, the mean intake of Fe from CF (4·6 mg/d) was close to the Indian DRI for Fe (5 mg/d) and approximately three times higher than that in the CG infants (1·5 mg/d). Our study design did not allow randomisation and the measurement of Fe status in the CG at 6 months of age. We, however, assume that the CG infants had comparable Fe status at 6 months of age with the IC infants at age 6 months and were mainly Fe-replete because the vast majority was exclusively breast-fed (94 %) and had food introduced after 6 months of life (mean age of food introduction: 6·6 months). Exclusive breast-feeding up to 6 months of life is associated with better Fe status in infants as it provides highly bioavailable Fe^(34)^ and reduces diarrhoeal diseases (and other infections) that accompany early introduction of CF, particularly in resource-limited areas where water and sanitation facilities are inadequate^(35)^. We therefore assume that the better Fe indices (Hb and sTfR) as well as the lower prevalence of anaemia, ID and IDA in the IC at 12 months of life compared with the CG are mainly due to the additional Fe from the study cereal reducing the risk of a decline in Fe status from 6 to 12 months of age in the IC. In our study, this is further supported by the higher needs of Fe supplements in the CG compared with the IC (twenty-one *v.* seven infants). Fe stores present at birth are mainly accumulated during the last 10 weeks of gestation and cover Fe requirements during the first 4–6 months of age in healthy term infants^(36)^. After this period, CF needs to provide sufficient Fe. However, home-made non-fortified CF in lower-middle income countries often have low Fe concentration and bioavailability^(5,37)^ and do not cover the high Fe needs during rapid growth in infancy^(38)^; hence, infants develop ID and IDA in late infancy when CF are introduced. This is confirmed by the low Fe intake from CF in the CG infants in the present study (1·5 mg/d) and their increased rates of ID (40 %) and IDA (26 %) at 12 months of age.

Fe bioavailability is an important aspect of Fe-fortified infant cereals as cereals contain phytate, which is the most important Fe absorption inhibitor, binding non-heme Fe in an insoluble complex in the intestine, making it unavailable for absorption^(39)^. For our rice-based infant cereal, we, however, assume little influence of phytate as polished rice has a low phytate concentration comparable with refined wheat flour, which is frequently used for infant cereals^(40)^. We estimate intermediate bioavailability of 10–15 % from the studied infant cereal as proposed by WHO for cereal foods with some vitamin C^(41)^. We assume that this bioavailability is substantially higher than that from home-made non-fortified CF consumed in the CG not containing vitamin C, a strong enhancer of Fe absorption^(39)^. Assuming 10–15 % Fe bioavailability, the infants in the IC absorbed between 0·38 and 0·56 mg Fe/d from the study cereal which is more than half (52–78 %) of their basic daily physiological Fe requirements needed for growth and coverage of basal Fe losses (0·72 mg/d)^(42)^. As for every fortified food vehicle, achieving good organoleptic properties and high Fe bioavailability can be challenging^(43)^. The Fe compound selected should be the one with the highest relative bioavailability that causes no sensory changes when added to the food vehicle. FeF used in our study cereal is one of the compounds recommended for the fortification of CF^(41)^. This recommendation is based on the compound’s good sensory properties and on results from isotope studies that reported similar Fe absorption values for FeF and ferrous sulphate (FeSO_4_) (relative bioavailability of FeF with regard to FeSO_4_ as standard: 100)^(44,45)^. Unlike FeSO_4_, FeF caused few or no sensory changes in stored cereal products or colour changes in porridges made by adding hot milk or water to the infant cereals^(46)^. Together with ferric pyrophosphate and electrolytic Fe, FeF is the most commonly used Fe compound to fortify CF. FeF should, however, be preferred due to its better bioavailability^(43)^.

The aetiology of anaemia is multifactorial^(2)^, and our data using SF < 12 μg/l to define ID indicate that other factors than Fe alone contributed to anaemia in our population group. Inflammation and infections can cause anaemia and reduce the sensitivity of SF to detect ID but can be ruled out as substantial contributors in our study population because we did not include sick infants and the CRP concentrations were low in both groups. Nutritional deficiencies in folate, vitamin A and vitamin B_12_ also contribute to anaemia via ineffective erythropoiesis^(47,48)^ and might have played a role in the anaemia of the infants in CG as they did not receive additional amounts of these micronutrients and were at risk for deficiencies. Vitamin B_12_, which is not found in any plant foods, seems to be of particular interest as a substantial proportion of Indian women are vegetarians (30 %)^(6)^, and the concentration of vitamin B_12_ in breast milk was associated with maternal diet^(49–51)^ as well as with maternal and infant status of vitamin B_12_^(52)^. Hence, low maternal vitamin B_12_ status may have put the infants in our study at risk for vitamin B_12_ deficiency at 6 months of age due to an inadequate supply of vitamin B_12_ during exclusive breast-feeding. Two recent studies reported high prevalence of vitamin B_12_ deficiency in Indian infants (44 and 57 %)^(53,54)^, and vitamin B_12_ status was an important predictor for Hb concentration^(55)^. By receiving additional vitamin B_12_ from the study cereal, infants in the IC were likely to improve their vitamin B_12_ status which may have had a positive impact on their Hb concentration. Indeed, the vitamin B_12_ intake from CF in the IC (0·18 µg/d) was much closer to the Indian B_12_ DRI (0·20 µg/d) than in the CG (0·08 µg/d). The concentration of vitamin A in breast milk has also been associated with maternal diet^(49)^, and high prevalence of vitamin A deficiency has been reported in Indian women and young children^(56)^. Our study infants were likely to be at risk for vitamin A deficiency as even the significantly higher intake of vitamin A in the IC (210 µg/d) compared with the CG (30 µg/d) was still below the Indian DRI for vitamin A (350 µg/d). For folate, we do not assume a substantial impact on Hb concentration in our study. We found no difference in intakes between the two groups at 12 months of age. This is likely because the traditional complementary feeding in India is rich in green leafy vegetables, which are exceptional sources of folate. Folate deficiency in breast-fed Indian infants from low- to middle-income communities was reported to be low (6 %)^(57)^, and folate concentrations in breast milk are maintained even when the mother is deficient in folate and are unaffected by maternal folate supplementation^(58)^.

In our study, infants in the IC had a statistically significantly higher Bayley-III scores for language and motor development than infants in the CG, who had significantly higher prevalence of ID and IDA than the IC infants. This suggests that by preventing the decline in Fe status in the IC, the fortified cereal helped the infants in the IC to make use of their full neurodevelopmental potential. Several previous studies reported detrimental motor and cognitive development in infants with ID or IDA^(8–12,59)^, which can impair development in infants via altered myelination of white matter, changes in monoamine metabolism in the striatum and functioning of the hippocampus^(60)^. In children 4–23 months of age, studies on Fe supplements (≥10 mg Fe/d) and fortification do however not provide conclusive evidence for a beneficial effect of Fe on the cognitive and motor development^(16–19)^. We found only one other study assessing the effect of a fortified infant cereal on motor development outcomes, in which mainly Fe-depleted infants receiving a multi-fortified maize-based cereal had better Fe status and parent-assessed gross motor milestones than their counterparts in the control group^(14)^. To our knowledge, our study is the first study assessing the social-emotional and adaptive behavior of infants in a study with fortified cereals using the Bayley-III scores and also the first data for Indian infants, in general, using this tool. Previous studies using different tools for the assessment of infant behaviour and other vehicles than cereals to provide Fe reported a beneficial effect of Fe on social interaction, irritability and orientation engagement^(13,61)^. It is likely that the favourable Bayley-III scores in our study are not only a result of the additional Fe but also of the other essential micronutrients as well as essential fatty acids provided by the infant cereal. In our study, complementary feeding with the fortified cereal resulted in significantly higher intakes of several key nutrients for brain development in the IC *v.* CG (except folate and vitamin D). In our intake calculations, we did not consider nutrient intake from breast milk consumption which was perhaps higher in the CG as indicated by the breast-feeding frequency data. However, with the limited volumes of breast milk consumed in older infants and the fact that higher breast-feeding frequency does not automatically imply consumption of higher volumes of breast milk, that is, higher nutrient intake, we assume little influence of the different breast-feeding frequencies on the differences in nutrient intake between the two groups observed at 12 months of age. Furthermore, the energy intake from CF was the same in both groups (2030 kJ/d in IC *v.* 1986 kJ/d in CG) indicating that it is unlikely that the CG infants consumed much more breast milk than the IC infants in addition to the CF. Our data suggest that although all Bayley scores for both groups are in the normal range, providing essential macro- and micronutrients helps the infants to better explore their full development potential.

Studies with Fe-fortified cereals in infants using a classical RCT design are of potential ethical concern because (1) in the control group, Fe is withheld from anaemic and/or Fe-deficient infants or infants highly susceptible to ID during a critical period of rapid growth when insufficient intake of Fe might have an irreversible negative impact on the infant’s development and cognition and (2) severe and moderate anaemic infants in the intervention group likely need higher dosages of Fe to recover their Fe status than conventional cereal fortification can provide. We therefore chose a design with a static observational group which did not withhold provision of Fe from anaemic and/or Fe-deficient infants or infants at risk for ID in a CG, and we did not include severe or moderate anaemic infants in the IC. This allowed us to assess specific real-world effectiveness data with high public health significance as non-anaemic or mildly anaemic infants account for approximately 60 % of the Indian infant population (31 and 29 %, respectively)^(6)^ and would benefit most from a food-based intervention that is more cost effective and sustainable than Fe supplementation. On the other side, our design did not allow us to randomise the infants; hence, unknown confounders might have influenced our results. This limitation was partially overcome by having infants in the static observational group that were matched on certain key characteristics with the IC. We analysed only one 24-h recall and were therefore not able to capture day-to-day or seasonal variations. Nevertheless, we think that the recall provides some valuable data to corroborate the differences in micronutrient intake between the two study groups. Our data cannot be extrapolated to more Fe-depleted infants with moderate and severe anaemia, who are likely to need pharmacological doses of Fe to recover their Fe status. Further research is needed to investigate the magnitude of the beneficial effect for other Fe compounds than FeF and different dosages of Fe.

In conclusion, our findings show that infants consuming a fortified rice-based cereal providing a low to moderate dose of Fe had better Fe status and favourable development scores for language, motor, social-emotional and adaptive behaviour than their peers not consuming the fortified cereal. The consumption of the study cereal was safe, supported age-appropriate growth without increasing weight-for-age and weight-for-length *Z*-scores and substantially increased the intake of key macro- and micronutrients important for infant development. In a setting where micronutrient deficiencies are prevalent, the improvement of nutritional status and development is most likely an interplay between several micronutrients and a multi-fortified CF can be an efficacious approach to reduce the risks of deficiencies in infants. Our findings emphasise how crucial it is for an infant 6–12 months of age to receive CF providing sufficient bioavailable Fe and other micronutrients in order to reduce the risk of ID and IDA, and in order to live up to their full development potential. IDA can be an obstacle to national development not only causing enormous health costs but also serious economic consequences^(62)^. Our model on social costs using the Hb difference found in the present study (9·7 g/l) indicated a tremendous reduction in Disability Adjusted Life Years and production losses which illustrate the massive potential of fortified infant cereals to contribute to better national development in India by preventing ID and IDA.
