# Micronutrient-fortified infant cereal improves Hb status and reduces iron-deficiency anaemia in Indian infants: an effectiveness study – Corrigendum

**DOI:** 10.1017/S0007114521002142

**Published:** 2021-09-28

**Authors:** Shally Awasthi, Narayan U Reddy, Monjori Mitra, Shweta Singh, Sanjeev Ganguly, Ivana Jankovic, Dominik Grathwohl, Colin I. Cercamondi, Apurba Ghosh

The authors apologise for an error in Table 3 The geometric mean for Serum ferritin (ng/ml) was incorrectly given as 40.0 when the correct value is 36.0. All other values remain the same. The corrected Table is given below.

**Correct Table 3.** Iron status indices and C-reactive protein concentration of infants in the intervention group at 6 and 12 months of age and in the static cross-sectional control group at 12 months of age. (Mean values and standard deviations; geometric mean and -1 standard deviation and +1 standard deviation)

Parameter	IC (*n* 64)				CG (*n* 80)		
	6 months		12 months		12 months		
	Geometric Mean	−1 SD, +1 SD	Geometric Mean	−1 SD, +1 SD	Geometric Mean	−1 SD, +1 SD	P*
Hemoglobin (g/l)							
Mean	113·4		118·1		109·5		<0·001
SD	8·7		10·2		16·4		
Serum ferritin (ng/ml)	36·0	16·4, 78·7	27·0	13·4, 54·4	20·3	7·5, 55·0	0·085
Serum soluble transferrin receptor (mg/l)	1·68	1·22, 2·31	1·70	1·19, 2·43	2·07	1·29, 3·33	0·014
Serum C-reactive protein (mg/l)	0·46	0·16, 1·34	0·84	0·20, 3·60	0·77	0·17, 3·39	0·741

CG, static cross-sectional control group; IC, intervention group; SD, standard deviation.

* *P*-value for between group comparison (ANCOVA model correcting for sex, exclusively breastfeeding until 6 months of age (Y/N), Kuppuswamy socioeconomic status^(1)^, birth weight, gestational age, and study site).

